# Congenital Telangiectatic Erythema: Scoping Review

**DOI:** 10.2196/48413

**Published:** 2023-10-05

**Authors:** Magda Sara Wojtara, Jayne Kang, Mohammed Zaman

**Affiliations:** 1 Department of Human Genetics University of Michigan Medical School Ann Arbor, MI United States; 2 Department of Health Sciences Queen's University Kingston, ON Canada; 3 Department of Biology Stony Brook University Stony Brook, NY United States

**Keywords:** rare diseases, rare disease, artificial intelligence, AI, dermatology, dermatologist, DNA repair, teledermatology, systematic review, erythema, deoxyribonucleic acid, bloom syndrome, postnatal growth deficiency, immune abnormality, cancer, oncology, DNA mutation, heredity

## Abstract

**Background:**

Congenital telangiectatic erythema (CTE), also known as Bloom syndrome, is a rare autosomal recessive disorder characterized by below-average height, a narrow face, a red skin rash occurring on sun-exposed areas of the body, and an increased risk of cancer. CTE is one of many genodermatoses and photodermatoses associated with defects in DNA repair. CTE is caused by a mutation occurring in the *BLM* gene, which causes abnormal breaks in chromosomes.

**Objective:**

We aimed to analyze the existing literature on CTE to provide additional insight into its heredity, the spectrum of clinical presentations, and the management of this disorder. In addition, the gaps in current research and the use of artificial intelligence to streamline clinical diagnosis and the management of CTE are outlined.

**Methods:**

A literature search was conducted on PubMed, DOAJ, and Scopus using search terms such as “congenital telangiectatic erythema,” “bloom syndrome,” and “bloom-torre-machacek.” Due to limited current literature, studies published from January 2000 to January 2023 were considered for this review. A total of 49 sources from the literature were analyzed.

**Results:**

Through this scoping review, the researchers were able to identify several publications focusing on Bloom syndrome. Some common subject areas included the heredity of CTE, clinical presentations of CTE, and management of CTE. In addition, the literature on rare diseases shows the potential advancements in understanding and treatment with artificial intelligence. Future studies should address the causes of heterogeneity in presentation and examine potential therapeutic candidates for CTE and similarly presenting syndromes.

**Conclusions:**

This review illuminated current advances in potential molecular targets or causative pathways in the development of CTE as well as clinical features including erythema, increased cancer risk, and growth abnormalities. Future studies should continue to explore innovations in this space, especially in regard to the use of artificial intelligence, including machine learning and deep learning, for the diagnosis and clinical management of rare diseases such as CTE.

## Introduction

Congenital telangiectatic erythema (CTE), also known as Bloom syndrome (BS), is a rare autosomal recessive disorder characterized by impaired DNA repair and increased susceptibility to cancer. As the name suggests, it is a condition characterized by visible, small, and linear broken blood vessels. In addition to this, photosensitivity is a very common feature in individuals with CTE. CTE was first described by David Bloom in 1954 upon observing several pediatric patients with similar physical features and cancer susceptibilities. In the 1960s, CTE was identified as a genetic disorder caused by loss-of-function mutations in the *BLM* gene and subsequent impairments in genomic repair mechanisms. Since then, research has largely been focused on understanding the biomolecular basis of symptom development and improving diagnostic methods for this disorder. Given the potential for cancer development, early detection is of crucial importance. Recently, artificial intelligence (AI) and machine learning advancements have resulted in more efficient identification of patterns of disease occurrence, management considerations, and the potential of aiding in finding novel therapeutics for screening [1]. These advancements may also eventually contribute to a greater understanding of CTE.

CTE is typically inherited in an autosomal recessive pattern where both parents are heterozygous carriers of the mutated *BLM* gene [2]. Existing knowledge on CTE suggests that mutations in RecQ helicases such as *BLM* result in accelerated aging symptoms and cancer incidence [2]. CTE has been most commonly observed in populations of Ashkenazi Jewish descent [3]. It has also been observed in consanguineous families. There are several dermatologic syndromes with similar presentations such as Rothmund-Thomson Syndrome, Erythropoietic Protoporphyria, and Cockayne Syndrome [3]. Rothmund-Thomson Syndrome in particular is also due to a mutation in a RecQ helicase [2]. Future studies, aimed at improving clinical management of CTE, will likely address how to differentiate between these similarly presenting syndromes.

CTE is characterized by a wide range of symptoms affecting multiple physiological systems. Common physical manifestations include short stature, delayed growth and puberty, a skin rash that occurs with sun exposure, in addition to a butterfly-shaped facial rash across the nose and cheeks. Common immunological manifestations include an increased susceptibility to infections and a higher risk of developing various cancers. The development of the erythematous skin rash upon sun exposure has been associated with a higher risk of developing squamous cell carcinomas [4]. CTE has also been associated with reduced fertility in women and infertility in men. While there are no known genetic differences in the *BLM* gene that lead to the development of heterogeneous symptoms, several cases of external genetic or environmental factors have been explored [5]. Our current understanding of the progression of CTE and its different presentations is limited given the rarity of the condition. A majority of current literature has elucidated key clinical features due to the willingness of current patients to participate in case studies and other forms of research.

## Methods

### Search Strategy

A literature search was conducted on PubMed, DOAJ, and Scopus using search terms such as “congenital telangiectatic erythema,” “bloom syndrome,” and “bloom-torre-machacek.” Due to limited current literature, studies published from January 2000 to January 2023 were considered for this review.

### Study Selection Process

The references of included articles were subsequently screened for potential addition. After we determined which papers were relevant according to title and abstract screening, full-text articles were obtained. These full-text articles were then screened and checked for inclusion and exclusion criteria. The data analysis plan and inclusion and exclusion criteria were established before screening to minimize potential biases. Two authors (MSW and JK) independently screened titles, abstracts, and full-text articles for potential inclusion in this review. Any discrepancies or disagreements were resolved and decided by the additional author (MZ). Ultimately, 278 records were screened, 121 were sought for retrieval, and 47 were assessed for eligibility.

### Inclusion and Exclusion Criteria

**Figure 1:**
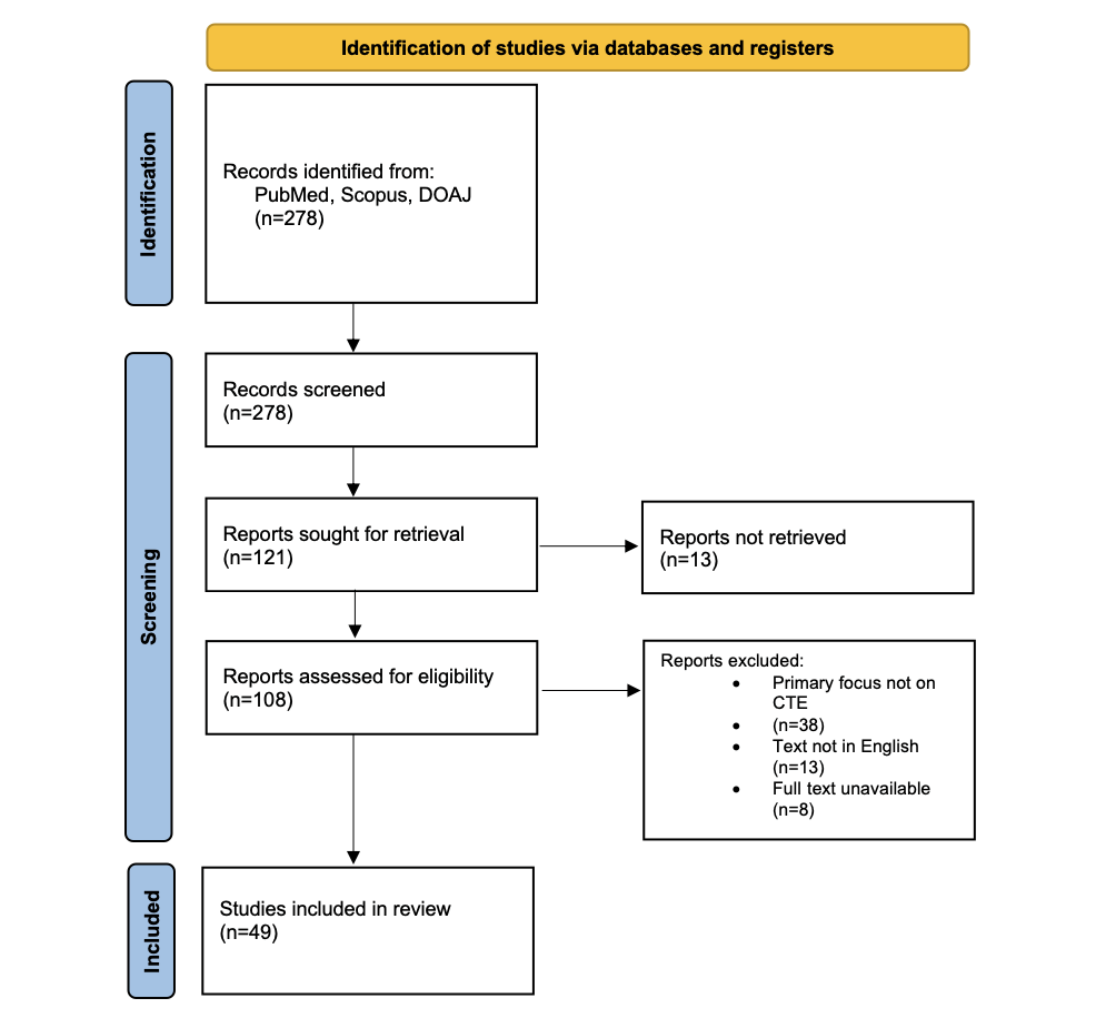
PRISMA (Preferred Reporting Items for Systematic Reviews and Meta-Analyses) 2020 flow diagram for the identification of studies [7].

All studies included in this review were published in English. Clinical trials were excluded from this review. Our criteria for inclusion were (1) relevance to CTE, (2) publication in English, and (3) full-text availability. Studies were further evaluated regarding demographic features, different clinical presentations, and management of CTE. The literature search yielded 278 studies, of which 47 initial studies met the inclusion criteria for further analysis. Any duplicates were removed, and papers that were not in English were also excluded from this review. After looking through the references of included studies, 2 additional sources were added for inclusion in the review and thus 49 studies were analyzed (Figure 1).

## Results

### Overview

This systematic review draws from 49 recently published articles on CTE heredity, CTE clinical presentation, management of CTE, and recent advancements. These studies were published in various countries including Australia, Canada, the United States, the United Kingdom, Saudi Arabia, Poland, Morocco, Japan, and China. These were analyzed after screening relevant literature. Due to the limited availability of research on rare diseases such as CTE, many of these articles have been published within the past 2 decades. The current literature focused on several main subject areas such as heredity, clinical manifestations, and management of CTE. A systematic review of our findings has been outlined in the following sections, which have been organized based on these themes. A summary of the studies used and examined, including country, year of publication, and key takeaways, are shown in Tables 1-3.

**Table 1:** Summary table for included studies focused on heredity.

Authors (years)	Characteristics	Summary
Ababou (2021) [7]	Patient data obtained from Bloom Syndrome Registry 1954-2018	Other than Ashkenazi Jewish descent, populations with high consanguinity rates have a higher prevalence of BS^a^.
Arora et al (2014) [3]	Patient data obtained from Bloom Syndrome Registry 1954-2009	BS shares similar clinical features to Rothmund-Thomson Syndrome, Erythropoietic Protoporphyria, and Cockayne Syndrome.
Bythell-Douglas and Deans (2021) [8]	Patient data obtained from cell cultures and from animal models	When the BS Complex is not present, the synapse is more likely to occur with the wrong sequence, which will lead to translocation and rearrangement.
Cunniff et al (2017) [9]	Patient data obtained from Bloom Syndrome Registry 1954-2017	The absence of functional BLM protein causes chromosome instability and excessive homologous recombination.
Enomoto (2001) [10]	RecQ family helicase biochemical assays	BLM is necessary for DNA replication—reserved in ND10s and is involved in a DNA surveillance mechanism operating during the S Phase.
Kaur et al (2021) [11]	Study data from yeast, Drosophila, mouse, and human	*BLM* has tumor-suppressing and pro-oncogenic characteristics, the paper focuses on the conditions under which the gene shows each of these characteristics.
Maciejczyk et al (2017) [12]	Study data from yeast, Drosophila, mouse, and human	The role of cellular redox alternations in the BS phenotype points to elevated superoxide dismutase activity and more production of reactive oxygen species in BS.
Mo et al (2018) [2]	RecQ family helicase biochemical assays	Mutations in RecQ helicases (including *BLM* that causes the BS) result in autosomal recessive syndromes characterized by accelerated aging symptoms and cancer incidence.
Monnat (2010) [13]	RecQ family helicase biochemical assays	Human RecQ helicases in cellular DNA metabolism need further research by studying conditions such as BS and how the acquired loss of RecQ function may provide new opportunities to improve cancer therapy.
Nakayama (2002) [14]	RecQ family helicase biochemical assays	*BLM* is identified as a caretaker-type tumor suppressor protein and a gatekeeper class of tumor suppressor proteins.
Vallance and Ford (2003) [15]	Data collected from genetic testing of carriers	A common mutation has been identified that involves the deletion of six bases and insertion of seven bases (2281 delATCTGAinsTAGATTC, abbreviated *blmAsh*) and leads to early termination of the BLM gene product.

^a^BS: Bloom syndrome.

**Table 2:** Summary table for included studies focused on clinical manifestations.

Authors (year)	Clinical manifestations
Bouman et al (2018) [16]	BS^a^ with a lack of sun-sensitive facial erythema
Giordano et al (2016) [17]	BS with erythema, telangiectasia, proportionate dwarfism, and increased risk of internal cancers. Cellular or genetic defects: quadriradial chromosomes; cells sensitive to ionizing radiation and alkylating agents
Capell et al (2009) [18]	BS with increased risk of cancer, ultraviolet hypersensitivity, hyper- and hypopigmented skin changes, decreased subcutaneous fat, immune deficiency, anemia, increased susceptibility to type II diabetes mellitus, severe growth retardation, and death by the age of 30 years usually due to cancer
Diaz et al (2006) [19]	BS with altered carbohydrate metabolism (however, small cohort, n=11)
Klein and Günther (2021) [20]	BS with photosensitivity and symptoms of immunodeficiency such as more frequent respiratory and gastrointestinal infections
Maciaszek et al (2020) [21]	BS with Wilm tumor—the most common childhood kidney cancer
Martin et al (2010) [22]	BS with growth abnormalities, hematopoietic defects, mutagen sensitivity, and cancer predisposition
Schierbeck et al (2019) [4]	BS with severe photosensitivity, poikiloderma, and erythematous telangiectasia, skin cancer, other malignancies of the upper and lower gastrointestinal and urinary tract
Prime et al (2001) [23]	BS with leukemia or lymphomas or solid tumors
Prokofyeva et al (2013) [24]	BS with epithelial carcinomas like breast cancer (not just lymphomas and leukemia)
Taylor et al (2019) [25]	BS with congenital abnormalities, pancytopenia, and cancer proneness

^a^BS: Bloom syndrome.

**Table 3:** Summary table for included studies focused on management.

Authors (year)	Clinical management suggestions
Balajee (2021) [26]	Using RecQL4 as a novel cancer therapeutic target
Ben Dhia et al (2023) [27]	Using dose reduction, regardless of chemotherapy type
Campbell et al (2018) [28]	Using dose reduction regardless of chemotherapy type
Frances and Cordelier (2020) [29]	Using cytidine deaminase as a novel cancer therapeutic target
Jastaniah (2017) [30]	Using rituximab-based chemotherapy protocol
Karalis et al (2011) [31]	Using greater awareness and patient education
Mojumdar (2020) [32]	Using RecQ as a novel cancer therapeutic target
Shen et al (2012) [33]	Using first-apparent cutaneous changes in the face
Walsh et al (2017) [34]	Using effective cancer screening

### Heredity of CTE

CTE is a rare genetic disorder usually found in an autosomal recessive pattern. Patients with CTE develop higher risk factors such as extremely high cancer rates up to “150 to 300 times” [8]. This is mostly due to the development of carcinogenesis in CTE-affected cells that may have laid the preset conditions for the development of multiple types of cancers. CTE is caused by a mutation in the *BLM* gene, which is responsible for the DNA repair enzyme RecQL3 helicase [3]. The mutation causes this essential repair enzyme to falter in its ability to repair and dispose of abnormal cells. The CTE complex consists of several protein components that function independently but in the grand scheme of DNA repair, and are crucial for rapid homologous recombination as well as the exchange of sister chromatids that carry the design information for CTE [10]. This uncontrollable flaw in the design information replication is what leads to genomic issues and the rise of abnormal conditions and cells. The development of the erythematous skin rash, a characteristic feature of CTE, upon sun exposure has also been associated with a higher risk of developing squamous cell carcinomas [4].

It has been observed that these proteins that are essential for DNA replication interact with RecQ homologs that function in a pathway that instigate the transition from DNA replication checkpoint to homologous recombination [11]. This leads to genomic instability that leads to the development of cancers such as lymphoma and leukemia which are prevalent in patients with CTE. With the formation of these many cancers, it has been noted that there is an increase in copy number, transcript, and protein levels which along with the fact there is a lack of wild-type *BLM* has increased the sensitivity of chemotherapeutic agents and has been labeled as pro-oncogenic. Therefore, there is a possible indication that BLM may act as a tumor suppressor [14]. This is also possible because within the *BLM* gene, resides the RecQ homologs, in particular, the RecQL4 helicase acts as a tumor suppressor. The RecQ homologs also act in the unwinding of intermediates of recombination, which prevents the unwanted execution and its defamation leading to the rise of unwanted genomic anomalies [35]. Various sources agree that there is a relationship between CTE and the rise of oncogenic conditions within these patients. Namely, this finding has been corroborated through the Bloom Syndrome Registry. This is an ongoing digital registry that serves as a surveillance mechanism to observe the effects of CTE over time [9]. Individuals with CTE are at a greater risk of complications such as various cancers, chronic obstructive lung disease, and diabetes [9]. Due to these complications, individuals with CTE stand to benefit greatly from improvements in screening and early detection.

Current literature suggests that CTE is more prevalent in certain ethnic groups than the others. Patients with CTE are mostly of Ashkenazi Jewish descent. Patients with CTE display certain physical phenotypes such as narrow facial features, elongated limbs, and several dermatologic complications including photosensitivity, poikiloderma, and telangiectatic erythema [3]. About 1 in 100 people with Ashkenazi Jewish heritage have the common founder mutation *blmAsh*, and there are also recurrent founder mutations among other ethnicities. Multiexonic deletions, nonsense mutations, frameshift mutations, and missense mutations have all been reported [13]. These essential processes occur because helicases use the energy of adenosine triphosphate hydrolysis to separate double-stranded nucleic acids [12]. Other phenotypes present in CTE are endogenous reactive oxygen species overproduction and impairment of mitochondrial homeostasis. Excess activity of antioxidant enzymes and an insufficient amount of low molecular weight antioxidants indicate new pharmacological strategies for patients having CTE [12].

### Clinical Presentations of CTE

CTE presents itself in a variety of symptoms and signs, some of which lead to further complications, and the studies that focused on clinical presentation displayed many similar trends and ideas with respect to how the syndrome manifests itself in the body. Most of the time, though not always, BS presents with sun-sensitive conditions, such as resulting facial erythema [16]. One case study showed some degree of phenotypic variation in facial erythema clinical presentations in CTE [16]. It is important for studies to establish the different possible clinical presentations of CTE in order to prevent delays in screening and early detection. However, photosensitivity and specifically facial erythema does remain one of the most common presenting signs [17]. This dermatologic feature is still one of the main components of initial diagnosis and screening for CTE. Altered carbohydrate metabolism is also very common in CTE and is often present from childhood [19]. BS dwarfism is not believed to be related to growth hormone deficiency or malabsorption, so the basis for growth restriction in CTE is unknown. It is believed that some of the variabilities in clinical manifestations are due to different pathomechanisms. A recent paper has suggested that the different pathomechanisms may be dependent on the number of micronuclei and the activity of BLM1 helicase and that interferon induction is possibly promoted or inhibited [20]. Other studies have suggested that because CTE is a constitutional chromosomal instability, it can result in a variety of syndromes including growth abnormalities, hematopoietic defects, and cancer predisposition [22].

BS also brings upon a predisposition for internal cancers, especially Wilm tumor, a common childhood kidney cancer [21]. Multiple articles present that factors such as chromosomal instability and mutations in the tumor suppressor genes in BS can cause a predisposition to lymphomas, leukemia, breast cancer, skin cancer, and oral cancer. An early paper demonstrated that patients with CTE were predisposed to either leukemia or lymphoma [23]. A more recent meta-analysis revealed that the BLM defect common in CTE not only increases the risk of leukemia or lymphomas but also epithelial carcinomas like breast cancer [24]. Most of the risk comes from when the signaling pathways of the ReqQ helicases are disrupted, which makes it similar to Werner and Rothmund-Thomson syndromes. Still, CTE brings on various results besides cancer, including immune deficiency, growth abnormalities, and susceptibility to diabetes mellitus.

### Management of CTE

The management of CTE and CTE-related cancers is presented with a unique set of challenges that is inherent to the impaired DNA repair mechanisms and subsequent DNA instability. Three studies describe the DNA-damaging effects of chemotherapy and radiation, and the associated risk of developing secondary cancers or myelodysplasia [27,28,36]. In such cases, chemotherapy dosage and duration reduction are recommended for individuals with CTE [27,36]. A study also suggests the benefit of broad-spectrum sunscreen with an SPF of at least 30 and the use of gamma-globulin infusions for reducing the frequency and severity of CTE-related infections [36].

With limited available treatments, several novel therapies are underway for more effective management and potential treatment. Two studies investigating novel therapies for CTE-related cancers suggest the use of RecQ helicases as a potential therapeutic target [26,32]. The catalytically active domains of RecQ helicases are described as a potential direct and tumor-specific target for small molecule inhibitors to be administered in conjunction with chemotherapy [32]. A case study in Saudi Arabia describes a successful treatment of CTE-related lymphoma in an 11-year-old patient with rituximab-based chemotherapy [30]. This demonstrates a safe treatment alternative for mature B-cell lymphoma in patients with CTE [30]. Another study describes potential therapeutic strategies through the modulation of cytidine deaminase (CDA) activity [29]. CTE is typically characterized by CDA deficiency, which has been observed to induce replicative stress [29]. The study presents CDA manipulation as a promising strategy for cancer therapy; however, greater research is warranted to better understand the role of CDA in oncogenesis [29].

Several studies emphasize the need for greater awareness and screening strategies for effective diagnosis [31,33,34,37]. A study discusses the use of candidate gene sequencing as a helpful tool for achieving a genetic diagnosis in children with distinct phenotypes [37]. However, the study also discusses the need for greater emphasis on the causative genetic mutations in phenotype-based diagnoses [37]. Another study summarizes the dermatological manifestations of inherited cancer and indicates cutaneous changes in the face as one of the most common early indicators of genetic syndromes with malignancies [31]. Furthermore, this study emphasizes the need for greater discussion and awareness among clinical practitioners, particularly dermatologists, for the effective diagnosis and treatment of those affected by CTE [31].

## Discussion

### Principal Results

CTE, also known as BS, is a rare genetic disorder that affects several physiological systems. CTE arises from mutations in the BLM gene which encodes the DNA repair enzyme RecQL3 helicase [3]. RecQL3 is a multidomain enzyme that constitutes the Bloom Syndrome Complex (BSC) and is able to repair double-stranded breaks in DNA [6,38]. BSC plays an integral role in the homologous recombination of DNA during DNA repair. Without a properly functioning BSC, chromosomal synapsis is more likely to occur with incorrect sequences, resulting in elevated levels of recombination-mediated insertions, deletions, and chromosome rearrangements. More than 60 different CTE-associated mutations have been identified within the BLM gene, including nonsense, missense, and exon-skipping mutations [6]. All forms of nonsense mutations have been associated with the absence of a C-terminal nuclear localization sequence, a C-terminal ssDNA annealing domain, and a portion of the helicase and RNaseDC domain, leading to improper localization of the enzyme [6]. A frameshift mutation that involves the deletion of 6 bases and the insertion of 7 bases (2281 delATCTGAinsTAGATTC, abbreviated *blmAsh*) has been associated with early termination of the *BLM* gene product and accounted for 97% of CTE alleles in patients of Ashkenazi Jewish descent [15]. Notably, CTE has an estimated incidence of 1 in 48,000 in the general population, while an estimated incidence of 1 in 120 has been identified in patients of Ashkenazi Jewish descent [39]. However, CTE has also been observed in consanguineous families [7]. The *BLM* gene is inherited in an autosomal recessive pattern, which means that an individual must inherit 2 copies of the mutated gene, 1 from each parent, in order to develop the condition [3].

CTE presents a range of clinical manifestations and impacts multiple systems in the body. As previously discussed, the absence of BSC has several detrimental effects on homologous repair such as reduced accuracy of gene regulation, decreased resection, and increased nuclease-mediated crossovers [38]. Such decreases in regulation fidelity during meiosis lead to reduced fertility in women and infertility in men [40]. In immune cells, the increased levels of incomplete recombination are known to stimulate the release of immunostimulatory DNA, inducing autoimmunity [41]. Associated symptoms include increased susceptibility to infections and sustained inflammation. In epithelial cells, genomic rearrangements from BSC infidelity have been associated with cancer initiation and progression [18]. In addition, the alterations in cellular redox regulatory mechanisms have been associated with elevated levels of superoxide dismutase activity and increased generation of reactive oxygen species in patients with CTE [12]. Given the breadth of physiological systems affected by the dysfunctional enzyme, CTE is characterized by a wide range of symptoms including growth deficiency, sun sensitivity, and predisposition to diabetes and cancer [25]. The severity and variability of CTE symptoms can vary widely among affected individuals, even among those with the same underlying genetic mutation. Some individuals may experience a milder form of the condition with only a few clinical manifestations, while others may have more severe and life-threatening complications. For instance, variations in immunological abnormalities have suggested a potential role in the frequency of infections in the affected individual [36]. Much research remains to elucidate the specific factors involved in the variation of clinical manifestations. CTE shares several clinical similarities with other chromosomal instability disorders such as Fanconi anemia and ataxia-telangiectasia [25]. These disorders are characterized by defective DNA repair and increased chromosomal instability, leading to similar clinical features, such as increased susceptibility to cancer and other complications [25]. Nonetheless, each disorder has distinct clinical and genetic features that differentiate them from one another.

### Implications

AI, including machine learning and deep learning types, are interesting areas of future study. There is limited current literature due to its recent advancements and it will likely be some time before it is applied specifically to CTE. Other rare diseases have already benefited from the use of machine learning to establish more efficient identification of patterns of disease occurrence, management considerations, and the potential of aiding in finding novel therapeutics for screening [1]. However, it is important to note that these advances are not a silver bullet solution and still require additional research inputs in order to provide accurate outputs.

AI and machine learning are harnessed by today’s modern health care systems to improve medical image processing, disease prediction and prevention, and hospital operations [42]. AI can also have use cases in all stages of drug development including discovery, preclinical stage, and clinical stage. For instance, it can contribute to initial validation, progression modeling, and diagnostic imaging. Through incorporating these approaches, better precision and effectiveness can be possible. AI and machine learning systems can allow health care providers and researchers to further optimize and accelerate the timeline for the diagnosis, treatment, and management of diseases [43]. For example, a recent study showed a use case that analyzed brain function and structural imaging data to determine whether a person with Huntington disease will receive a clinical diagnosis within 5 years (pre-Huntington disease) [44]. Especially in the case of rare diseases such as CTE, technological advances currently under development may be able to address many current challenges.

### Limitations

Our study has several limitations. First, we focused predominantly on existing peer-reviewed literature and were thus limited to drawing conclusions from case studies and existing information. Given the rarity of this condition, there were a limited number of publications that met our inclusion criteria despite broadening the time range. Since our review only included peer-reviewed scientific literature, however, some studies could be missed from nonmedical journal sources. Second, we did not assess in depth the intersection of any potential comorbidities that patients with CTE may commonly have. There were limited findings on patient attitudes toward different clinical management strategies for CTE or their experiences with disease progression. Another limitation is that this review does not include differences across intersectional demographic groups. Specifically, there is limited literature examining clinical presentation in skin of color dermatologic patients.

### Conclusions

BS, also known as CTE, is a rare autosomal recessive disease and as a result, there is a limited, but growing, body of scientific literature over the past 2 decades. Between 2000 and 2023, we identified 49 studies with a specific focus on heredity, clinical manifestations, and management of CTE. The incidence of CTE is unknown; however, the Bloom Syndrome Registry and initiatives within several rare disease organizations hope to help provide a clearer picture of key molecular markers and expand genetic testing access. Furthermore, using AI and machine learning platforms can help further elucidate potential therapeutic candidates and disease screening and progression. Diagnosis of CTE currently involves the identification of characteristic clinical features and molecular testing to identify changes to the *BLM* gene [45]. BS's clinical features include telangiectatic erythema and growth abnormalities. Key changes to the *BLM* gene, based on current literature, include an inverse relationship with chromosomal instability. Chemotherapy dosages for individuals with CTE should be reduced due to an increased likelihood of developing severe infections.

Overall, we were able to show what advancements have been made in understanding the heredity, clinical manifestations, and management of CTE. We have concluded that future studies should focus on answering several gaps identified in this review including which sequences in the *BLM* gene should be looked at as a marker for CTE in genetic testing. Additionally, future studies should continue to develop best practices for physicians consulting patients who have CTE. We conclude that CTE can also be challenging to diagnose due to a lack of access to genetic testing, low awareness of this rare condition, and other biological variations. Patients with CTE may also require alterations to typical chemotherapy or targeted immunotherapies in the treatment of cancer. Therefore, this topic is currently of great relevance for both oncologists and dermatologists. Continued efforts to widen information on CTE will support the development of a more comprehensive understanding of the clinical manifestations, heredity, and clinical management of CTE. Additional research will help to improve subsequent usage of AI to streamline candidate therapeutic selection, genetic testing, and clinical management of CTE.

## Appendix

Multimedia Appendix 1 Preferred Reporting Items for Systematic reviews and Meta-Analyses extension for Scoping Reviews (PRISMA-ScR) Checklist.

## Abbreviations

AI: artificial intelligence
BS: Bloom syndrome
BSC: Bloom Syndrome Complex
CDA: cytidine deaminase
CTE: congenital telangiectatic erythema
